# Achievements and Challenges of Genomics-Assisted Breeding in Forest Trees: From Marker-Assisted Selection to Genome Editing

**DOI:** 10.3390/ijms221910583

**Published:** 2021-09-30

**Authors:** Sunny Ahmar, Paulina Ballesta, Mohsin Ali, Freddy Mora-Poblete

**Affiliations:** 1 Institute of Biological Sciences, University of Talca, 1 Poniente 1141, Talca 3460000, Chile; sunnyahmar13@gmail.com; 2 The National Fund for Scientific and Technological Development, Av. del Agua 3895, Talca 3460000, Chile; 3 Department of Forestry and Range Management, University of Agriculture Faisalabad, Faisalabad 38000, Pakistan; mohsinali_uaf@hotmail.com

**Keywords:** Bayesian genomic prediction, BLUP, CRISPR/Cas9, GRF-GIF, forest tree improvement

## Abstract

Forest tree breeding efforts have focused mainly on improving traits of economic importance, selecting trees suited to new environments or generating trees that are more resilient to biotic and abiotic stressors. This review describes various methods of forest tree selection assisted by genomics and the main technological challenges and achievements in research at the genomic level. Due to the long rotation time of a forest plantation and the resulting long generation times necessary to complete a breeding cycle, the use of advanced techniques with traditional breeding have been necessary, allowing the use of more precise methods for determining the genetic architecture of traits of interest, such as genome-wide association studies (GWASs) and genomic selection (GS). In this sense, main factors that determine the accuracy of genomic prediction models are also addressed. In turn, the introduction of genome editing opens the door to new possibilities in forest trees and especially clustered regularly interspaced short palindromic repeats and CRISPR-associated protein 9 (CRISPR/Cas9). It is a highly efficient and effective genome editing technique that has been used to effectively implement targetable changes at specific places in the genome of a forest tree. In this sense, forest trees still lack a transformation method and an inefficient number of genotypes for CRISPR/Cas9. This challenge could be addressed with the use of the newly developing technique GRF-GIF with speed breeding.

## 1. Introduction

Forest trees are a main source of wood, energy, pulp for paper, soil protection, correction of erosion problems and silvopastoral plantations, among other things. In this sense, important efforts in forest tree breeding have been carried out to improve traits of economic interest, to select trees (or hybrids) adapted to new habitats, or to generate trees that are more tolerant to various biotic and abiotic stresses. From the point of view of wood productivity, conventional breeding programs have continuously been improving traits related to throughput, such as wood volume, tree height, diameter at breast height, stem straightness, survival and wood quality, under various environmental conditions [1,2,3,4,5,6,7].

Conventional forest tree breeding considers phenotypic evaluations based on kinship relationships between individuals to identify trees that are superior in a trait of interest. The Henderson mixed-model method has been traditionally used to determine the genetic merit of trees and best linear unbiased prediction (BLUP) has been the standard prediction method [8,9]. The genetic merit is predicted according to the degree of kinship between the trees of a breeding population under the assumption that individuals with a common ancestor are phenotypically similar and share loci of interest. Due to the long rotation time of a forest plantation and the resulting long generation times necessary to complete a breeding cycle, the advent of molecular marker technology along with traditional phenotypic selection has led to the development of marker-assisted selection (MAS) [10]. With MAS, important advances have been made in forest selection and breeding. In this method, the genotypic values of individuals are predicted by considering the effects of selected markers, which predictions are more accurate for traits controlled by few quantitative trait loci (QTLs), each of which controls a relatively large proportion of the phenotypic variation [11]. On the other hand, the development of high-throughput genotyping techniques has allowed the use of more precise methods for determining the genetic architecture of traits of interest, such as genome-wide association studies (GWASs) and genomic selection (GS). For example, GS is a method that uses molecular markers distributed throughout the genome, such as single-nucleotide polymorphisms (SNPs), which is preferred for complex traits that are affected by many genes and are strongly affected by the environment [12,13,14].

Both in traditional selection methods and in GS, the predictive accuracy of the phenotype of an individual depends on how accurate the estimation of the genetic relationships between individuals is [15,16,17]. The predictive power of a GS model can be greater if the individuals which are used as references to estimate the additive effects (of the loci) are genetically related to the individuals whose phenotype is to be predicted [17,18,19]. In this context, to make good use of GS, several genetic factors that are intrinsic to the study population should be considered [20], such as linkage disequilibrium (LD) patterns, the genetic structure of the population and genetic diversity, among others [21,22,23,24] in order to optimize the rates of genetic gain. Kinship relationships derived from pedigrees represent the (theoretical) proportion of the genome that is shared between individuals. This makes the relationship matrix generated by genealogical background an unbiased estimate of the kinship relationships between the genes that control the phenotype [15,25]. However, in some populations, it is not possible to perform a good reconstruction of the genetic structure that defines the population (for example, due to pollen contamination or in open pollination assays), which leads to lower precision in the estimation of genetic parameters [26]. Even events in the evolutionary history of the population could be ignored, which would make it difficult to accurately estimate the genetic parameters that are key in the selection of superior trees. In this sense, kinship estimates based on genomic regions are a better approximation of the real proportion of the genome that two individuals share. Currently, kinship between individuals is determined based on SNPs or haplotypes [27,28,29]. According to Edwards (2015) [27], haplotypes can establish more precise and reliable genealogical relationships than a relationship matrix made purely from SNPs because they allow us to examine the identity by descent that exists among the individuals of a population. Interestingly, haplotypes have been used in GS models, covering extensive regions of the genome of some annual plants (such as wheat and maize), which has increased the predictive power of complex phenotypic traits [30,31,32,33,34]. Interestingly, the haplotype approach can be especially beneficial for predicting traits with a relatively low heritability. Some authors suggest that these results can be explained by the fact that the use of haplotypes in GS allows access to heritable genetic components that cannot be detected by SNPs [31,35]. Despite these possible benefits demonstrated in silico and experimentally, there is still little information on the use of haplotypes in GS, especially in forest trees. In *Eucalyptus globulus* Labill., Ballesta et al. (2019) [36] emphasized that a GS model that uses haplotypes as predictor variables can increase the ability to predict traits with low genetic control by up to 21%. In addition, Mora-Poblete et al. (2021) [3] showed that the genomic prediction of some metabolites and components of leaves could become more accurate with the use of haplotypes in *Eucalyptus cladocalyx* F. Muell.

On the other hand, with the advent of genomic editing, great opportunities are visualized in terms of the future development of plant science, including in forest trees and in the rapid remodeling of crops. Genome editing is a relevant, versatile and preferred tool for the improvement of annual crops, as well as for functional genomics [37]. Great advances have been made in gene editing techniques, such as those using zinc finger nucleases (ZFNs), transcription activator-type effector nucleases (TALENs) and clustered regularly interspaced short palindromic repeats (CRISPR) associated with the Cas9 and Cpf1 proteins [38,39,40,41,42]. Among the various genome editing tools, CRISPR has become the most popular, which has helped to clarify the genomic structure and its role in plants, for example, the transcriptional control of Cas9 and Cpf1, the monitoring of a genetic locus, the mechanism and control of the promoter activity of a gene and the alteration and detection of epigenetic behavior at SNPs as found through GWASs [38,43,44]. This review describes various methods of forest tree selection assisted by genomics, from MAS to genomic editing and the main technological challenges, highlighting the achievements and advances in research at the genomic level.

## 2. Conventional Forest Tree Breeding and Marker-Assisted Selection (MAS)

In conventional forest tree breeding, phenotypic evaluations are performed and kinship relationships are determined to identify the trees that are superior in a trait of interest [26,45,46,47]. In general, evaluations are carried out in progeny trials, such as half and/or full siblings, clones, hybrids, or a combination there of them [48,49,50]. Phenotypic selection has contributed significantly to the increase in genetic gains in various forest species around the world, such as *Eucalyptus*, *Pinus*, *Populus*, *Picea* and *Cryptomeria* [51,52,53,54,55]. However, the maintenance of progeny trials, for example, involves a significant investment of resources, intensive logistics and long selection cycles before usable results are seen [10,56,57].

Parental genotypes are evaluated by the throughput of their progeny, such that allelic combinations that generate offspring with superior throughput are considered genetically superior and are put into successive breeding cycles. Therefore, an accurate estimation or prediction of the “breeding value” of an individual is required. On the other hand, it is common that it is complicated to analyze the results of a forest competition trial because (1) usually only a subset of the parents is represented in each progeny trial; (2) the parents are represented in different number of progeny trials; (3) the trials are evaluated at different ages of the trees; and (4) the kinship background between individuals may be wrong due to the management of a seed orchard or even due to inbreeding processes [26,58]. The historical innovation for the breeding value estimation model taken into account the effects caused by the genotypes as random effects instead of fixed [59,60,61,62]. This analytical method, known as best linear prediction (BLP) or best linear unbiased prediction (BLUP), allows the maximization of the precision or the correlation between the predicted and real values for genetic merits, which includes the pedigree information of the individuals evaluated [63]. In this sense, the use of the Henderson mixed-model method (based on BLUP) has traditionally been used to determine the genetic merit of trees and it is considered a standard prediction method [8,9]. The genetic merit is predicted according to the degree of kinship between the trees of a breeding population under the assumption that individuals with a common ancestor are phenotypically similar and share loci of interest. However, conventional breeding has allowed significant genetic gains in different forest species [4,49,64], even when it presents clear disadvantages, such as the long generation time required to complete a breeding cycle [10,65]. The advent of molecular marker technology, in combination with traditional phenotypic selection, has led to the development of marker-assisted selection (MAS) [10]. With MAS, important advances have been achieved in forest selection and breeding. MAS predicts the genetic merit of the trees by considering the effects of a group of selected markers (considering their statistical significance) and it has become the preferred way to improve traits controlled by few loci, each of which controls a relatively large proportion of the phenotypic variation [13,66].

## 3. High-Throughput Genotyping Techniques Enable Different Fields of Studies on Plants

Molecular markers have been widely used in genetic and plant breeding studies [67,68,69,70]. SNPs, for example, are abundant in plant genomes and their usefulness as genetic markers has been well established in the last decade [71]. For this reason, SNP markers have been applied to various areas of knowledge, such as forensic science and diagnostics in humans, aquaculture, livestock marker-assisted selection, agricultural crop breeding and conservation studies [72,73,74,75,76].

In 2011, the number of sequenced plant genomes doubled compared to the previous decade (http://phytozome.net, accessed on 1 September 2021), which was due to the increasing throughput of sequencing methods. Second- and third-generation sequencing platforms, such as Illumina’s various platforms, 454 pyrosequencing (Roche), SOLID (Invitrogen) and Ion Torrent (Invitrogen), have the ability to obtain results from many sequences, which can be used to discover new molecular markers in a viable way and at low cost [77,78]. These techniques have been used on a large scale for the discovery of SNPs in representative sets of individuals of various species of plants, such as rice [79], wheat [80] and different trees [81,82]. The discovery of SNP markers has made important contributions to advancements in various fields of study, such as genomics, transcriptomics and population genetics, such as for mapping QTLs. Through knowledge of the genome and the physical locations of these polymorphisms, DNA arrays have been made to type SNPs of different species, such as cacao [83], cherry [84], wheat [85] and rice [86].

Given the need for molecular tools that support genomic studies of species of interest in forestry, different SNP arrays that are transferable between taxa of the same genus, such as *Populus*, *Eucalyptus*, *Quercus*, *Picea*, *Araucaria*, *Pinus* and *Pseudotsuga* have been developed [87,88,89,90,91,92,93] (Table 1). For example, a 50 K Axiom array, developed from the genome of *Picea abies* (L.) H. Karst., can be applied to at least four species of *Picea*, while EUChip60K has been applied to more than 14 taxa of the genus *Eucalyptus* [81]. The numbers of SNPs detected in coniferous and hardwood species have been relatively similar, but conifers tend to have considerably larger genomes [94], which implies that SNP arrays developed in conifers could have less genome coverage than those developed for broadleaves.

## 4. Genomic Selection/Prediction, an Extension of BLUP Methods to Maximize the Predictive Power of Traits of Interest

After the emergence of molecular markers on a large scale and the decrease in costs associated with genomic tools, breeders began to use DNA markers to support selection cycles. In general, genomic-assisted breeding can be based on MAS or genomic prediction/selection [105,106,107]. The efficiency of each of the methods varies according to the genetic architecture underlying the trait under study. MAS estimates the genotypic value of an individual from the effects of selected molecular markers and it has greater effectiveness in phenotypic traits that have an oligogenic genetic architecture [107]. GS is a preferred method when studying complex traits that are affected by a large number of genes and are highly influenced by the environment. In the case of forest trees, several QTLs related to various traits of interest have been identified and mapped in association studies, such as growth [108,109,110,111], flowering components [112,113,114], pulpability [115,116,117] and wood properties [110,118,119,120,121]. However, effectively, the application of MAS in traits with polygenic inheritance has been limited. In this sense, it is worth mentioning that the important traits of a forest plantation, such as growth, are controlled by many genes that each contribute little to the phenotypic variation [51,109].

GS was first proposed by Meuwissen et al. (2001) [105] in the context of animal breeding as a method to specifically increase the efficiency of dairy cattle breeding programs. GS was born as an alternative to conventional BLUP based on pedigree information. Unlike in MAS, in GS the effects of thousands of molecular markers are predicted simultaneously, even though these are not individually significant for a trait. According to Daetwyler et al. (2013) [122], GS can widen the range of genetic gain since the individual genetic merits are estimated with greater precision. Although GS does not allow us to identify the function of a gene controlling a trait, the predictive models provide a short-term selection criterion of those individuals who have a better throughput. Moreover, GS has helped us understand the genetic architecture of phenotypic traits and even to implement ecological restoration and biological conservation plans [123,124]. The most widely known GS methods incorporate models derived from the so-called Bayesian alphabet: Bayes A, Bayes B, Bayes Cπ, Bayesian ridge regression (BRR), Bayesian least absolute shrinkage and selection operator (LASSO) [105,125,126] and the conventional methods of genomic BLUP (GBLUP) [127] and ridge-regression BLUP (RR-BLUP) [105]. RR-BLUP and GBLUP assume that the markers have the same variance and that each marker makes a small contribution to the prediction model (infinitesimal model). The prediction via GBLUP is performed similarly to that via BLUP, the difference being that the BLUP pedigree matrix is replaced by a relationship matrix constructed from molecular markers. RR-BLUP is a multiple regression method in which the markers are thousands of regressors that explain the variation in a phenotypic trait. In the context of the RR-BLUP method, the breeding (genomic) value of each individual is defined by the following formula [128]:

(1) $$GEBV_{j}=\overset{n}{\underset{i}{\sum }}Z_{ij}\;{\hat{m}}_{i}$$

where *n* corresponds to the total number of markers, ${\hat{m}}_{i}$ is the estimated effect of the *i*th marker and $Z_{ij}$ corresponds to the design matrix associated with the vector of the effects of the markers, which encodes the genotype of the *j*th individual at the *i*th marker. The abbreviation GEBV stands for genomic estimated breeding value.

In contrast, the Bayes A, Bayes B and Bayesian LASSO methods assume that each marker has its own variance, and the phenotypic variance is explained by loci with effects of different magnitudes [129]. These Bayesian methods are differentiated by the prior distributions that are established and the degree of fit chosen. A more detailed description of each method can be found in Heslot et al. (2012) [130] and De Los Campos et al. (2013) [131]. For example, the Bayesian LASSO method assumes that the effects of the markers are distributed a priori according to a double exponential (*DE*) distribution: pmi|λ,σε2=DE(mi|0,λσε2), where λ is a regularization parameter. The *DE* distribution generates a strong contraction (close to 0) to estimate the effects of the markers. BRR is a Bayesian method that is based on the model’s regressors (whether SNPs or other markers), which have a common variance ($\sigma_{m}^{2}$), so that those regressors with the same allelic frequency explain the same proportion of the additive variance and have the same contraction effect [132]. The a priori distribution of the marker effects (*m_i_*) is Gaussian, and Var(*m_i_*) takes a single value of $\sigma_{m}^{2}$. In the Bayes A model, it is assumed that each marker (*m_i_*) follows an independent normal prior distribution with mean 0 and variance $\sigma_{m_{i}}^{2}$, while the variance of each of them is assumed to be distributed as $\sigma_{m}^{2}|v,S^{2}\sim \chi^{-2}\left(v,S^{2}\right)$, where $S^{2}$ and v are the parameters for scale and degrees of freedom, respectively. On the other hand, the Bayes B and Bayes C methods include the parameter π, which corresponds to the probability that the effect of a marker is equal to 0. For the parameter π, an a priori distribution π ~ β (p_0_, π_0_) is assumed, such that p_0_ > 0 and π_0_ can take values between 0 and 1 [133]. In both Bayes B and Bayes C, the effects of the markers have an a priori normal mixture distribution, such that $m_{i}|\pi \sim \left(1-\pi \right)N\left(0,\sigma_{m_{i}}^{2}\right)+\pi N\left(0,\sigma_{m_{i}}^{2}=0\right)$. On the one hand, in the Bayes Cπ method, it is considered that all the markers have a common variance and this variance is distributed in a similar way as in the BRR method, while for the Bayes B method, the variance of the markers follows a similar distribution to that in Bayes A.

In practice, it is recommended to test all available GS methods [134], which should be contrasted in terms of their accuracy or predictive ability. However, if the researcher has an idea of how many loci explain the variation of a trait, he could use a particular method. For example, the Bayes B model bases its analytical assumptions on highly heritable traits and whose variation is explained by large-effect loci [134]. On the other hand, Bayes A represents an option for traits that are controlled by a moderate number of genes. Some studies have shown that Bayesian methods are usually more accurate than GBLUP when the training and validation populations are weakly related genetically [135,136].

With computational advancement and algorithms enhancements, artificial neural networks (ANNs) have emerged as an alternative statistical framework and have gained increasing interest in genomic studies [1,137,138,139,140]. This method can be particularly useful when the number of unknown variables is much higher than the number of samples (high-dimensional genomic information), since ANNs have the ability to capture non-linearities, adaptively [1,141]. In the context of plant/tree breeding, few approaches have included non-parametric approaches and non-linear functions based on ANN methods. Maldonado et al. (2020) [1], for instance, investigated several genomic selection models to predict several complex traits in breeding populations of *Zea mays* L. and *Eucalyptus globulus* Labill., including two Machine Learning (ML) methods, i.e., Deep Learning (DL) and Bayesian Regularized Neural Network (BRNN), both in combination with different hyperparameters. The results showed that DL had a superior performance than GBLUP, BayesA, BayesB, BayesCπ, BRR, BL, RKHS and BRNN, in terms of predictive ability for all traits (tree growth and stem quality-related traits in the case of *E. globulus* Labill.), confirming the importance of deep learning models in genome-wide studies and crop/tree improvement, which holds promise for accelerating breeding progress. Moreover, Pérez-Rodríguez et al. (2012) [142] found that BRNN and Radial Basis Function Neural Networks (RBFNNs) (non-linear models) had higher predictive accuracy for grain yield and days to heading in wheat and smaller predictive mean-squared error than Bayesian linear regression models. On the other hand, Zingaretti et al. (2020) [143] evaluated the predictive accuracy of linear and DL techniques in two important small fruits or berries: strawberry and blueberry (polyploid outcrossing species) and did not find an advantage of DL over linear model methods BL and BRR, except when the non-additive component (epistasis) was important. In fact, linear Bayesian models were better than convolutional neural networks for the full additive genetic architecture, whereas the opposite was observed under strong epistasis. Interestingly, Alves et al. (2020) [144] compared GBLUP with ANN in a simulated study considering different levels of dominance effects. They found that ANN had a higher prediction accuracy compared with GBLUP for traits with moderate narrow-sense heritability (h^2^ = 0.30) and dominance effects of 0 or 0.15. In this regard, Maldonado et al. (2020) [1] found that the DL approach outperformed GBLUP despite the low dominance effect, confirming that DL is a promising alternative tool for genomic prediction independent on the contribution of additive and/or dominance genetic effects.

In the context of forest tree breeding, GS was originally proposed for the analysis of complex traits such as tree growth and wood-related traits. The main parameter that reveals whether a GS model is adequate for the estimation of genetic merits is the precision and predictive power, which expresses the degree of correlation that exists between the genetic values predicted by the GS model and the adjusted phenotypic values (or adjusted breeding values) [145,146,147]. In the study by Beaulieu et al. (2014) [12], an accuracy of up to 0.435 was obtained in the GS models to predict traits related to wood properties in fir tree. In *Pinus pinaster* Aiton, Isik et al. (2015) [148] reported that even with a relatively low density of markers, the ability to predict traits related to growth can be moderate (0.43–0.49). The predictive ability of GS models can be increased using different methodological strategies. For example, Cappa et al. (2019) [149] utilized the single-step GBLUP (ssGBLUP) method, for growth and wood-quality traits in *Eucalyptus*, which consists of simultaneously considering genotyped and nongenotyped trees. According to the authors, the use of additional phenotypic information from nongenotyped trees provided greater predictive ability than GBLUP. Additionally, Ballesta et al. (2020) [150] showed that GS principles can be combined with the GWAS method to increase the ability to predict phenotypic traits with different genetic architectures in *Eucalyptus cladocalyx* F. Muell.

On the other hand, genomic tools have allowed access to heritable components that cannot be examined through genealogical relationships between individuals [26,151,152]. For example, Müller et al. (2017) [151] determined that the heritability of the diameter at breast height in a population of *Eucalyptus pellita* F. Muell was too low to calculate (extremely low) by the pedigree method; however, the genomic heritability based on SNPs reached a value of 0.55 (Bayes method B). Table 2 summarizes some of the studies on GS published in the last 5 years on different productive traits in trees.

## 5. Factors That Determine the Accuracy of Genomic Prediction Models in Forest Trees

Above, it was discussed how the accuracy of genomic prediction methods changes according to the analytical assumptions that support each method. However, genetic factors that are specific to breeding populations determine the effectiveness of genomic-assisted selection. The existing pattern of LD in a given breeding population is one of the main factors that determines the power of genomic tools to predict phenotypic traits because the extent of LD throughout the genome of a species determines the density of markers that is necessary for accurate prediction [23,24]. Strictly speaking, if the LD spreads across relatively large genomic distances, a lower density of markers could be necessary because it increases the probability of detecting markers that are in LD with QTLs. In contrast, if LD decreases within a relatively short genomic distance, a higher density of markers would have to be used to obtain a more accurate prediction.

The magnitude of LD in a population comes from the history and dynamics of the population [173]. In general, the LD in populations of cross-pollinating plants (such as most species of interest for forestry) decreases rapidly as the physical distance between markers increases because the number of effective recombinations is relatively higher than in self-pollinating species. For example, LD ceases to be significant at distances of 3000–6000 bp in natural populations of *Populus trichocarpa* Torr. & A.Gray ex Hook. [174] and within a distance of less than 1000 bp in *Pinus taeda* L. [175] while in self-pollinated species, such as soybeans and rice, the LD can be significant (in some populations) at interlocus distances of 10,000 bp and 25,000 bp, respectively (reviewed by [176]). Additionally, populations of forest species tend to be more heterozygous in their loci due to the reproductive mechanisms that these species have, since they have large effective size, high genetic diversity and low intrapopulation genetic differentiation [177,178].

Natural populations of forest species have lower LD values than cultivated and improved populations [151,158,179,180,181]. For example, LD in a natural population of *Populus* can decrease within 750–1000 bp [179,180], whereas in an improved population, LD can extend up to 2500 bp [181,182]. In the case of coniferous populations, the disequilibrium can decrease within much shorter genomic distances than for other tree species (<1000 bp). In the case of breeding populations of *Eucalyptus*, LD can decrease rapidly over 3000 bp or 25,000 bp [26,36,121,158,183], while in populations that have not been subjected to selection, the disequilibrium pattern can decrease within 500 bp [184].

Another factor that affects the accuracy of GS is the effective population size [17,185]. According to Grattapaglia and Resende (2011) [186], the effective size is inversely proportional to the level of accuracy of the GS model. For example, if it is desired to predict a phenotype controlled by 50 QTLs, with an effective size of 100 individuals and a density of 2 markers/cm, a predictive value of 0.36 is estimated, which can be increased to 0.8 if we have a density of 20 markers/cm. However, with an effective size of 10 individuals, under these same conditions, the predictive power can be increased from 0.73 to 0.88, which shows that small effective sizes allow us to increase the predictive power of a model, especially with a low density of markers.

In this same context, both in GS methods and in pedigree-based prediction, the predictive accuracy of the phenotype of an individual depends on how accurate the estimation of the genetic relationships between the individuals of the population is [15,16]. The predictive power of a GS model can be increased if the individuals who are used as references to estimate the additive effects of the loci (training population) are genetically related to the individuals whose phenotype is to be predicted (validation population) [17,18,19,187]. The genetic structure of a population is a factor that influences the degree of precision that a genomic prediction model can have [26,156,188,189,190]. The genetic structure of a population is defined by the degree of kinship that exists between the individuals who make up the population, which can be established by the genealogical background, or by genomic data. In the case of individuals who come from a natural population, the genetic structure can be given by the genealogical background and by the existence of subpopulations (genetically differentiated groups) within the same population. For example, Tan et al. (2017) [156] showed that the prediction of some traits in *Eucalyptus* hybrids could be favored by using a training population that is closely related to the validation population in terms of pedigree and population genetic structure. In contrast, if the training population is composed of individuals genetically distant from the validation population, the predictive ability can be reduced by up to 25%.

The determination of kinship relationships between individuals or organisms has been an important aspect in several fields of knowledge, such as forensic science, conservation genetics and animal and plant breeding [27]. Estimates of kinship between individuals are traditionally based on pedigree data, in which it is assumed that those individuals who act as founders of populations (for example, families) are not genetically related [191]. If the individuals of a population are genotyped, the polymorphic loci which are shared may be identical by state (IBS) or identical by descent (IBD), depending on whether these loci were inherited from the same ancestor. It is expected that individuals who share the same parental lines (full siblings) have on average 50% alleles that are IBD. However, the percentage of the genome they share is subject to variation due to random events that may occur. In this sense, genetic relationships based on pedigrees are arbitrary and theoretical; therefore, they are not necessarily a reflection of the real way in which genomes are inherited. Despite this drawback, pedigree data have been widely used to establish relationships between individuals of a population in quantitative genetics research [191]. Based on this problem, VanRaden (2008) [127] proposed making predictions via BLUP using molecular markers (SNPs) to determine kinship relationships within a group of individuals instead of by using the pedigree matrix (this method later came to be known as GBLUP). Additionally, Yu et al. (2006) [192] proposed using marker-based relationship matrices as covariates in GWAS models. SNP biallelic markers do not have enough information at the individual level, so some studies suggest that a high density of markers is required to establish reliable genealogical relationships [193,194]. Additionally, in populations with multiple founders, generations and few descendants, genotyping errors are difficult to detect [195,196]. An alternative for kinship analysis is the identification of haplotypes [27,28,29]. When two or more loci have a low probability of recombination between them, combinations of alleles called haplotypes are formed [197,198], which are genomic regions within a chromosome that tend to be inherited in joint form [199,200,201]. In this context, Edwards (2015) [27] proposed evaluating the kinship between individuals based on the construction of haplotypes present in the study population. According to this same author, these kinship relationships can estimate more precise genealogical relationships than the VanRaden relationship matrix [127]. If one wants to know the intrapopulation coancestry, it is most advisable to use haplotypes that are IBD. However, haplotypes can have both IBD and IBS relationships.

## 6. The Detection of Alleles in Narrow LD Allows the Optimization of the Accuracy of Genomic Prediction Models

Several studies have evaluated the predictive power of phenotypic traits in GS models that include haplotypes from SNP arrays [35,202,203,204,205,206]. One of the advantages of using haplotypes in GS is the ability to detect mutations [205]. According to Curtis et al. (2001) [207], when mutations have occurred, it is possible that the allele frequencies will remain (almost) unaltered. However, when haplotypes are analyzed, mutations in different loci tend to cause important changes in haplotype frequencies. Therefore, a QTL that is not in complete LD with an individual marker may be in complete LD with a specific haplotype. Moreover, haplotype-based approaches can include epistatic effects, in addition to additive effects [208,209], which is of interest in forest tree breeding (for example, in clonal selection and in the dissection of adaptive traits [4,210]. Additionally, the use of haplotypes, instead of individual markers, reduces the degrees of freedom in the prediction or genomic-association models, which contributes to greater accuracy in the detection of QTLs [192]. An also relevant aspect is the size of a haplotype found in a given population. The longer the haplotype (greater number of SNPs in LD), the fewer the effects that must be estimated, which leads to more precise estimates [211] and they are simpler to handle computationally.

The predictive power of haplotypes and molecular markers (individually) could depend on the trait that sought to be improved. In the context of animal breeding, some authors suggest that the haplotype approach may be especially beneficial for predicting traits of relatively high heritability [205,212,213]. However, in plants, prediction based on haplotypes has been especially beneficial for predicting low-heritability traits [31,33,34]. For example, Matias et al. (2017) [31] reported that the predictive accuracy of the models based on haplotypes was higher than the accuracy based on SNPs (not grouped into haplotypes) at predicting the yield of corn grains, but this result was not observed in the genomic prediction of plant height. According to these same authors, the yield of maize grains has low genetic control compared to the height of the plant. In the context of forest species, Ballesta et al. (2019) [36] showed that genomic prediction based on haplotypes can be an especially suitable approach for traits with low genetic control (h^2^ < 0.1) in *Eucalyptus globulus* Labill.

Villumisen and Janss et al. (2009) [35] showed, through simulated data, that genomic predictions based on haplotypes are especially beneficial for traits with low genetic control (relatively low heritability), in which haplotypes formed by five SNPs in LD have better goodness of fit and predictive power than models based on markers not grouped in haplotypes. According to [214] the use of haplotypes permits evaluations at the multiallelic level, which lead to a better representation of the variability associated with the traits with low heritability, which are generally controlled by several QTLs of relatively small effect. In this context, haplotypes would allow access to heritable components of certain phenotypic traits that cannot be captured by SNPs. The effectiveness of the haplotype approach in GS depends on how the haplotypes are defined in the study population [31,205]. For example, a haplotype can be defined according to a certain number of polymorphic loci, a defined size (genomic distance), or according to an LD threshold value. It is common for haplotypes to be defined by a certain number of SNPs [30,35]. Since this approach does not consider the LD between the markers and the historical recombination events of the population, some genetic background may not be considered in quantifying the variability between individuals [205].

Some studies suggest that GS based purely on molecular markers can lead to a loss of genetic variability, which leads to an increase in the rate of inbreeding [215,216,217,218]. For example, Rutkoski et al. (2015) [215] showed that the genetic gains for rust resistance in wheat obtained by GS and phenotypic selection are equivalent; however, GS generates a faster reduction in genetic diversity (per year) than phenotypic selection. In a simulated study, Lin et al. (2016) [216] reported that GS would double and triple the genetic gains of persistence and throughput of *Lolium perenne* L. with respect to phenotypic selection, respectively. However, GS led to a higher rate of inbreeding per selection cycle than phenotypic selection. This compromise between genetic gain and loss of diversity has not been a focus of study in forest species; however, it is also expected that GS can cause a significant loss of genetic diversity compared to traditional schemes that use phenotypic selection [219]. In this sense, several studies have proposed different strategies to establish a balance between genetic gains and post selection conserved diversity [183,219,220,221]. According to Daetwyler et al. (2015) [122], GS based only on SNPs could generate the loss of certain deleterious alleles (or those that apparently have no effect on the phenotype) from the population; however, haplotypes allow us to manage a selection based on alleles that do or do not have effects but remain in LD. In this sense, GS based on haplotypes could also contribute to good management of genetic resources in such a way that genetic gains could be obtained without sacrificing genetic variability.

Haplotype-based GS has been mainly implemented in agricultural crops and self-pollinated plants (for example, wheat; [33], where high LD values can be found throughout their genomes, which favors the identification of haplotypes in a population. In cross-pollinated plants, such as forest species, LD usually decreases within short genomic distances, which allows the identification of smaller haplotypes made up of fewer alleles. In this sense, the use of haplotypes in the GS of forest species could be restricted by the genotyping density of the population. On the other hand, Mora-Poblete et al. (2021) [3] showed that a low density of SNPs and consequently of haplotypes, could be compensated for by the combined use of GS and the principles of GWASs. In *E. cladocalyx* F. Muell, the authors reported that the genomic predictive ability of cyanogenic glycoside and anthocyanin contents, based on haplotypes, can be improved by the use of haplotypes significantly associated with these quantitative traits

## 7. Genome-Wide Association Studies (GWAS)

High-throughput genotyping technology and phenotyping platforms have enabled large-scale marker-trait association analysis, such as GWAS, to precisely dissect the genetic architecture of plant traits [222]. In trees, several studies have reported many putative genomic regions associated with variation of related-traits to tree phenology [223,224], wood properties [118,120,165,225,226,227,228,229], growth (i.e., wood volume, tree height and diameter; [108,111,117,120,226,230,231,232], resistance to pests and diseases [233,234,235,236], among others. For example, McKown et al. (2018) [223] implemented a GWAS analysis with the motivation to understand the molecular mechanisms of the variation in bud-break of flowers in *Populus trichocarpa* Torr. & A.Gray ex Hook. The authors identified ~30 polymorphisms within 16 annotated genes in the *Populus trichocarpa* genome, which were mainly associated with meristem growth, bud activation, cell expansion and proliferation, cold acclimatization and heat response. Recently, Elfstrand et al. (2020) [236] reported eleven marker-trait associations (MTAs) associated with the resistance to *Heterobasidion parviporum* in *Picea abies* (L.) H. Karst., which explained between 2 and 5% of the phenotypic variation of the studied trait. Additionally, the authors demonstrated that the *PaLAC5* gene, one of the candidate genes related to resistance to *H. parviporum*, could be involved in the development of the length of the lesion, as an induced defense response. Bai et al. (2019) [231] identified 29 nuclear collections from ~150 *Pinus massoniana* Lamb. trees, which were evaluated for different phenotypic traits. They found a large number of SNPs significantly associated with resin production, wood volume, tree height and diameter. In general, many marker-trait associations with relatively large effect have been detected for wood properties, disease resistance and phenology [237].

GWASs have been carried out considering different analysis strategies, which are often complemented with studies of gene co-expression networks, transcriptomic studies, heterologous expression assays, among others. For instance, Valenzuela et al. (2021) [111] identified ~90 SNPs and haplotype blocks associated with growth and stem quality traits in *E. cladocalyx* F. Muell under arid conditions, 11 of which were common between tree height, wood hardness and diameter; a result consistent with the trade-off between hydraulic safety and efficiency in *Eucalyptus* trees under drought conditions. Interestingly, the significant SNPs and haplotype blocks were located close to genes involved in the primary metabolism, biosynthesis of cell wall components and stress response genes, which could be related to the mechanisms of adaptation to stressful conditions. Lamara et al. (2016) [225] developed an integrative approach that involves association mapping results and co-expression networks in white spruce trees (*Picea glauca* (Moench) Voss). In this study, the authors tested SNPs into ~2000 candidate genes for statistical associations with microfibril angle, wood density, stiffness and ring width. The co-expression networks revealed complex interactions and pleotropic effects between genes involved in wood stiffness and microfibril angle. Baison et al. (2019) [238] performed a GWAS for several wood-related traits in *Picea abies* (L.) H. Karst., using ~170 K SNPs generated from exome genotyping of mother trees. The authors identified more than 50 SNPs associated with 39 candidate genes, of which their role in wood formation and tree growth has previously been recognized. Moreover, to understand the genetic mechanisms underlying wood anatomical and morphological traits in *Populus trichocarpa* Torr. & A.Gray ex Hook., Chhetri et al. (2020) [228] performed a functional enrichment analysis on coexpression nearest neighbors for gene models by the wood anatomical and morphological trait GWAS analyses. The results evidenced that the genes affecting cell wall composition and transport related genes were enriched in wood anatomy and stomatal density trait networks. Signaling and metabolism related genes were also common in networks for stomatal density. They concluded that the identified genes provide further insights into the genetic dissection of wood anatomical and morphological traits in *Populus*, which are important determinants of the suitability and sustainability of improved genotypes for lignocellulosic biofuel production.

Muchero et al. (2018) [234] employed a GWAS strategy to identify putative loci associated with the resistance to the invasive fungal pathogen *Sphaerulina musiva* in *Populus trichocarpa* Torr. & A.Gray ex Hook. About 90 SNPs encompassing 73 candidate genes were significantly associated to the number of cankers, number of cankers cm^−1^ and disease severity based on digital imagery. Interestingly, three loci were functionally validated by a transcriptomic study, allele analysis, binding assays and overexpression assays. Recently, Quan et al. (2021) [229] performed a combined approach that includes GWAS, transcriptomic analysis and transgenic experiments to dissect the genetic architecture of wood properties and photosynthesis in *Populus tomentosa* C.K. Schneid. The authors detected SNPs related to ~170 candidate genes for the studied traits, 74 epistatic relationships between the phenotypes and several pleiotropic loci. In addition, the heterologous expression of two pleiotropic genes in *Arabidopsis thaliana* (L.) Heynh. (i.e., *PtoMYB62* and *PtoMYB80*) demonstrated that these genes control the regulatory networks of photosynthesis and the components of the secondary wall, respectively, in *P*. *tomentosa*.

In terms of new challenges to implementing the integration of different OMICs technologies, Du et al. (2018) [239] discussed about the challenges and prospects of GWAS to improve wood properties in major timber species, including *Eucalyptus*, *Populus* and various coniferous species. The authors summarized the recent progress in GWAS-based functional genomics of these traits and concluded that the emerging high-throughput phenotyping technology will be broadly used in the future to collect data for quantitative studies of complex traits related to tree growth, adaptation, morphological and physiological traits.8. Genome editing in forest trees

The anticipated expansion of the population in the coming decades will significantly boost demand for forest products. Due to the long juvenile period and genomic complexity of forests trees, the genetic improvement via conventional breeding is laborious and time-consuming. Therefore, genetic modification (GM) provides the potential for transformation in shorter timeframes but is challenged by existing genetically modified organism (GMO) laws. Genome editing (GE), which may generate mutations in sites, allows for the fast implementation of specific changes and is less restricted worldwide than genetically modified technology [240,241]. Genome engineering in forestry is urgently needed given the rise in human activity and the effects of climate change (for instance, changes in rainfall distribution and the increase in severe droughts). Based on many field investigations, GE technology strengthens wood products from intensively cultivated planted trees. It may be especially relevant given the rapid increase in biotic and abiotic stress on forests [242,243].

GE techniques such as Zinc Finger Nuclease (ZFN) and transcription activator-like effector nuclease (TALENs) may be used to modify the genome. However, these methods are either labor demanding or prohibitively costly since the targeting mechanisms are all dependent on protein-nucleic acid interactions, necessitating creating a unique protein for each gene locus of interest [244]. Recent advancements in understanding prokaryotic adaptive immune systems provide another approach for genome editing named clustered regularly interspaced short palindromic repeats and CRISPR-associated protein 9 (CRISPR/Cas9). It is a highly efficient and effective genome editing technique that has been used to effectively implement targetable changes at specific places in the genomes of forest trees [245,246,247,248]. The Doudna group revealed the primary mechanism through which Cas proteins and CRISPR arrays functioned in 2012 [249]. CRISPR/Cas9 is a more versatile method among other genome engineering tools like Zinc Finger Nuclease (ZFN) and transcription activator-like effector nuclease (TALENs) [250].

**Figure 1:**
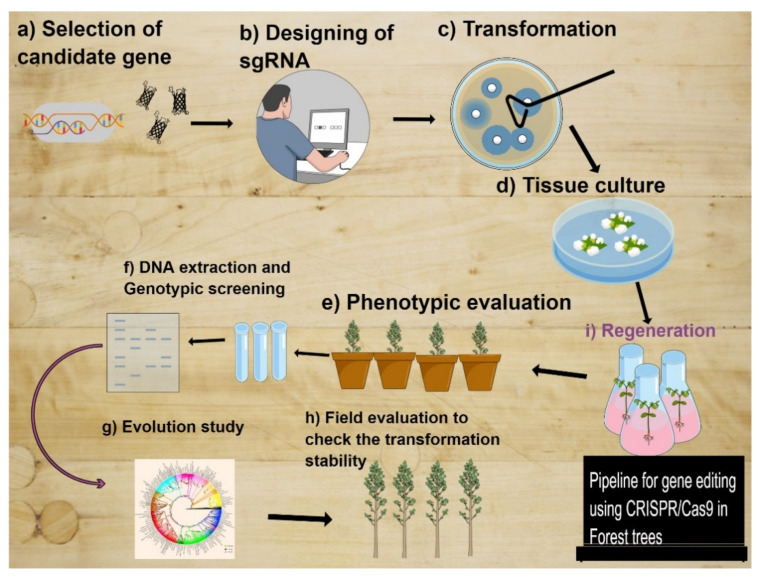
The pipeline of CRISPR/Cas9 to develop the transgenic plants in a forest tree. (**a**) Selection of candidate gene according to the trait under selection. (**b**) Design of the target on the gene sequence using any available online tool for sgRNA designing. (**c**) Construction of vector and transformation of cas9 vector into the explant through agrobacterium mediated transformation. (**d**) Develop the transgenic plants by tissue culture and regeneration of plant from the explant. (**e**) Regenerated plants are grown under glasshouse conditions to check the phenotypic according to the proposed objective. (**f**) Extract the DNA for genotypic analysis of plant carry mutation either homozygous or heterozygous or mutation didn’t occur. This step can be performed before phenotypic evolution. (**g**) On this step, check the evolutionary study if necessary, according to the trait under selection. (**h**) Choose the best plant based on phenotype as well on genotypic analysis for field evaluation.

This method enables editing single to many genes by knock-in or knock-out genes from the host genome. Thus, various traits may be introduced or metabolic pathways modified concurrently by inserting double-stranded breaks (DSBs) at multiple locations [251,252,253]. The other most important aspect about the use of site-directed mutagenesis in plants is that it is relatively inexpensive, effective and easy to apply compared to other techniques [246,254]. CRISPR was also successfully used by targeting potential development and biosynthesis pathway genes in grapes as well as in the tropical tree *Parasponia andersonii* Planch [255,256]. Although CRISPR/Cas9 applications are expected to begin with economically significant agricultural plants, the growing number of undescribed species whose whole genomes are being sequenced will enable the technique to be used more widely throughout the plant kingdom. The pipeline to adapt the CRISPR in forest tree is given in Figure 1.

Most significant progress has been achieved in woody species to date with poplar, which was the first stably transgenic tree to be genome-edited with high efficiency using the CRISPR/Cas9 system [257,258]. The first time CRISPR/Cas9 has been used for bi-allelic mutations in woody perennials [259]. Four members of the 4-coumarate: CoA ligase (*4CL*) gene family were targeted in *Populus* using CRISPR/Cas9 genome editing. One of the genes from the *4CL* family is known as the *4CL1*gene and it has been widely studied for its role in lignin production (Table 3). The lignin content of all edited transgenic plants was decreased by about 23%, with a corresponding reduction in the S/G lignin ratio of around 30% [257,259,260]. Furthermore, the CRISPR/Cas9 technology was also used to alter the genome of *Populus tomentosa* Carr. It was shown that a protospacer-adjacent motif (PAM). is followed by four guide RNAs (gRNAs) that target the phytoene desaturase gene 8 (*PtoPDS-8*) in poplar through Agrobacterium-mediated transformation and albino phenotype were observed in homozygous plants. Researchers found that mutation efficiency at these target locations was assessed to be 51.7% based on RNA-guided genome editing events and suggesting that CRISPR/Cas9 is efficient method to edit the genome of woody plants [248,259]. Most CRISPR studies have been addressed phenylpropanoid metabolism and/or cell wall properties in poplar. CRISPR-knock out (CRISPR-KO) of MYB transcriptive factors either raised the flow of phenylpropanoid (*PtoMYB156* and *PtrMYB57*) or reduced the flow (*PtoMYB115* and *PtoMYB170*), influencing the lignin deposition (*PtoMYB156* and *PtoMYB170*) [261,262]. CRISPR-KO mutants showed a brassinosteroids biosynthetic gene, which was similarly affected by secondary wall synthesis, indicating the involvement of brassinosteroids in the development of wood [263].

Additionally, CRISPR/Cas9 works very well and accurately in two poplar clones to produce *LEAFY(LFY)* and *AGAMOUS* (*AG*) mutations through a transgenic approach. A distinct mutation spectrum was observed *LFY* and *AG* in sgRNA-gene combinations. While an AG-sgRNA construct containing two sgRNAs produced comparable mutation spectra between two poplar clones, an LFY-sgRNA construct containing a single sgRNA produced substantially different mutation spectra between the same two clones [241]. Similar genetic studies were applied on *Eucalyptus* [264]. CRISPR Cas9 was used to produce transgenic plants in the *Eucalyptus* orthologue of *LFY* by converting a *Eucalyptus grandis* x *urophylla* wild-type hybrid and two Flowering Locus T (FT) overexpressing lines targeting the *LFY* orthologues of ELFY. The CRISPR-KO mutants achieved 100% transgenic insertion such as deletion, frameshift mutation and phenotypically transgenic plants as the absence of male and female gametes and indeterminacy in floral development due to floral alteration because of disruption of ELFY function. Similarly, Van Zeijl et al. (2018) [256] presented a quick and effective technique for *Agrobacterium tumefaciens*-mediated transformation and CRISPR/Cas9 mutation within 03 months in the fast-growing tropical tree species *Parasponia andersonii* Planch. They edited the four genes *PanHK4*, *PanEIN2*, *PanNSP1* and *PanNSP2* that regulate cytokinin, ethylene, or strigolactone hormonal pathways and, in legumes, perform important symbiotic activities. CRISP-KO mutants of *PanHK4* and *PanEIN2* reduced the procambium activity *and* disturbed sex differentiation, respectively. In contrast, CRISP-KO mutants of *PanNSP1* and *PanNSP2* were essential for nodule formation. Using CRISPR/Cas9, forest tree genomics may be taken to the next level by evaluating gene function and its role in adapting trees to their environment [247,265]. Multiple DNA repair pathways may be involved in CRISPR/Cas9-induced mutations, according to published tree studies. These studies suggest that the sequence context at or near the target sites may affect mutagenesis results. Available findings indicate persistent CRISPR-induced mutations and related phenotypes across many clonal generations enabling commercial production of elite trees propagated vegetatively [240,258,266].

Furthermore, in recent years, Landstrasse and Grosshansdorf (2019) [267] checked the efficiency of sgRNA in a poplar species (*Populus tremula* L.) using CRISPR/Cas9. They selected twelve genes for three distinct study areas, including *SOC1*, *FUL*, their paralogs, four *NFP*-like genes and *TOZ19*. The sgRNAs were created for editing with Cas9 nuclease and transferred into *P. tremula* L. and regenerated plants showed different types of editing, with single nucleotide insertions being the most common occurrence. They attempted to establish a correlation between genome editing and gRNA efficiency by evaluating the genome editing effort. They suggested that the GC content, purine residues in the final four nucleotides of the gRNA and an at least partially unpaired seed region all affected the gRNAs effectiveness for target cleavage [267,268]. Similar study has done an in *Pinus radiata* D. Don using CRISPR/Cas9. They explored the use of this system to edit the xylan 1 (*GUX1*) gene in *P. radiata* D. Don and showed genome editing using DNA and RNPs [269]. They concluded that CRISPR/Cas9 can generate biallelic and monoallelic INDELs in the coniferous tree *P. radiata* D. Don using DNA and RNPs, respectively. This study enables the use of genome editing in conifers to change the desired traits, or attributes, quickly.

Toxic diseases are causing just as much damage in forest plantations. They are a worldwide concern for forest ecosystems and must be handled as soon as possible. Ideally, CRISPR/Cas9 should be integrated into forest development projects to create more effective disease resistance methods for long-term forest sustainability [240]. For example, using CRISPR/Cas9 to combat Dutch elm disease (DED) pathogen *Ophiostoma novo-ulmi* is another intriguing potential, in which it has been demonstrated that these genes are excellent candidates for CRISPR/Cas9 gene editing to generate knockout mutants with decreased capacity to switch the DED pathogen throughout the elm tree life cycle [270,271]. Due to their lengthy vegetative life and poor seed laying rates, it is challenging to produce homozygous mutants via self-pollination in many forest tree species. Ding et al. (2020) reported a feasible method to decrease the incidence of chimeric mutant poplar trees with CRISPR/Cas9 with the second round of shoot regeneration utilizing leaves as the explants. A total of 15 transgenic plantlets were screened for homozygous mutants of *PdbPDS1*. Only one was found, which was confirmed by both phenotypic and genotypic analysis; in T0 generation, all transgenic plants were chimeric. Still, during the second round of shoot regeneration, about 27.0 percent or 19.1 percent of the regenerated shoots were homozygous mutants with or without kanamycin selection, respectively.

Despite the advancement in genome editing, CRISPR/Cas9 has limited use in the formation of transgenic forest trees. Recently, CRISPR/Cas12a is a newly developed novel CRISPR effector protein supporting the CRISPR/Cas to edit the larger genome fragments. Two popular species, such as *Populus alba* L. and *Populus glandulosa* Moench were subjected to CRISPR/Cas12a to achieve targeted mutations using three nucleases AsCas12a, AsCas12a and LbCas12a. It has been utilized in using CRISPR/Cas12 to knock off various targets of the *PDS* gene. AsCas12a is a more appropriate and efficient method to edit the big part of the genome at editing sites with the most remarkable mutation. For multi-gene knockout mutation in forest trees, the advantages of CRISPR/Cas12a for the creation of transgenic tree species are only further amended. A method for forest genetics for developing transgenic tree species will be provided using CRISPR/Cas12 [272]. There are still specific issues for the developing transgenic trees due to their longer life span and vegetative growth developmental stages. In woody perennials, CRISPR-based transformations in the behavior of trees to grow under different sets of environments, growth acceleration for the production of wood, nuts and barriers are much needed in the modern era [273].

## 8. GRF-GIF Chimeras Could Be Gamer Changer Tools in Forest Editing to Boost Tree Regeneration

Low plant regeneration efficiency and few transformable genotypes restrict the potential of genome editing to enhance the performance of crops and forest trees [264,266]. Genetic engineering of woody plants has many challenges, including poor transformation efficiency, a lack of knowledge on optimum expression cassettes and difficulty isolating clonal-modified plants [274]. Additionally, low regeneration efficiency limits the plant material from being transformed. Two independent investigations have showed that GROWTH-REGULATING FACTORs (GRFs) alone or when combined with GRF-INTERACTING FACTOR (GIF) may drastically increase tissue culture regeneration from diverse plant species. In terms of plant transformation and gene editing, GRF-GIF chimeras may be a game-changer for genome editing in dicot species and forest gene editing [275]. Debernardi et al. (2020) [276] demonstrated that the efficiency and speed of regeneration are improved in wheat, triticale and rice using a protein expression that combines GRF4 and GIF1; this leads to an increase in the number of transformable wheat genotypes. Moreover, a combination of GRF4–GIF1 and CRISPR/Cas9 genome editing produced 30 altered wheat plants with mutations in the gene Q (*AP2L-A5*). Finally, they demonstrated that a dicot GRF–GIF chimera increases regeneration efficiency in citrus, indicating that this approach may be applied to other dicot crops [277].

**Figure 2:**
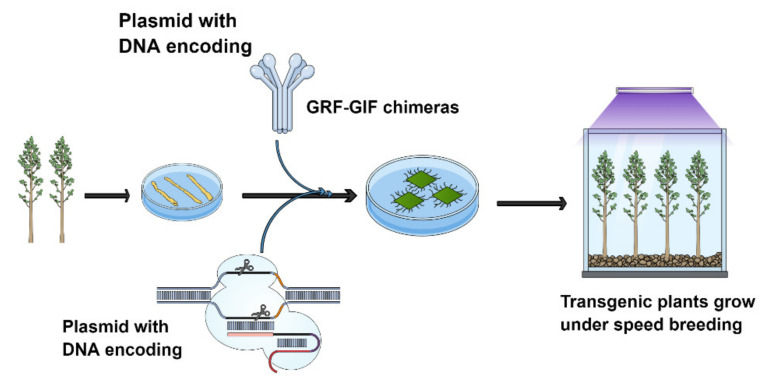
Construction of CRISPR/Cas9 vector with GRF-GIF chimeras to edit the genomes of tree breeding populations. After constructing the vector, do the transformation as per available protocol in the lab with different genotypes. The regenerated plant should be grown under speed breeding protocol to boost the growth period for the phenotypic and genotypic analysis to do further investigation quickly modified by [275].

According to the study performed by Debernardi et al. (2020) [276], we believe that this strategy will also be helpful in forest gene editing, especially in those trees in which there is no stable transformation method yet and no optimize genotype for transformation through CRISPR/Cas9. For this method, we can prepare a CRISPR/Cas9 construct with the combination of dicot GRF–GIF chimera for any forest tree (e.g., poplar) for the Agrobacterium transformation to generate the transgenic plant. By using this strategy, the researcher can try different types of genotypes to check the regeneration efficiencies. This hypothesis was already proved in wheat, rice and citrus and regeneration efficiencies were high compared to control for detail [275]. The transformation protocol in wheat was reduced and faster with the GRF4-GIF1 chimera five weeks than normal ones. It is suggested that transformation will also be faster in trees. After generated the transgenic tree plants, then grow the transgenic plants under speed breeding protocol to boost up the growth of the plant for the screening of the homozygous or heterozygous plant as well phenotypic evolution [278,279,280,281]. Speed breeding is a breeding method that reduces the generation time and speeds up breeding research programs by a considerable amount. Due to the 22-h photoperiod and the temperature control, the generation time has been significantly shortened. As a result of speed breeding, spring wheat (*Triticum aestivum* L.), durum wheat (*T. durum* Desf.), barley (*Hordeum vulgare* L.), chickpea (*Cicer arietinum* L.), pea (*Pisum sativum* L.) and canola (*Brassica napus* L.) may produce up to six generations each year instead of two or three under regular glasshouse circumstances (Figure 2). For detail about speed breeding, see [278,279,280,281]). Using these strategies, a researcher can save time to edit the genome of forest trees by CRISPR/Cas9.

**Table 3:** List of tree species successfully transformed genetically and potential candidates to be genome-edited with CRISPR/Cas9 and CRISPR/Cas12.

Tree Species	Method	Targeted Gene	Transformation Method	Findings	References
*Populus*	CRISPR/Cas9	*4CL1*, *4CL2*, *4CL5*	AT	Role in lignin production. The lignin content of all edited transgenic plants was decreased by about 23%, with a corresponding reduction in the S/G lignin ratio of around 30%.	[259]
*Populus tomentosa* Carr	CRISPR/Cas9	*PtoPDS*	AT	Chlorophyll biosynthesis, albino phenotype	[248]
*Populus tomentosa* Carr	CRISPR/Cas9	*MYB57*, *MYB115*, *MYB156*, *MYB170*	AT	Ectopic deposition of lignin, xylan and cellulose during secondary cell wall formation	[261]
*Populus tomentosa* Carr	CRISPR/Cas9	*BRC1-1*, *BRC2-*	AT	Secondary wall synthesis, which is responsible the involvement of brassinosteroids in the development of wood	[263]
*Populus tremula × P. alba*	CRISPR/Cas9	*AG1*, *AG2*, *LFY*	AT	A distinct mutation spectrum was observed *LFY* and *AG* in sgRNA-gene combinations	[241]
*Parasponia andersonii* Planch (tropical tree)	CRISPR/Cas9	*EIN2*, *HK4*, *NSP1*, *NSP2*	AT	Regulate cytokinin, ethylene, or strigolactone hormonal pathways and, in legumes, perform important symbiotic activities	[256]
*Populus tremula* L.	CRISPR/Cas9	*SOC1*, *FUL*, *NFP TOZ19*	AT	GC content, purine residues in the final four nucleotides of the gRNA and an at least partially unpaired seed region all affected the gRNAs effectiveness for target cleavage	[267]
*Pinus radiata* D. Don	CRISPR/Cas9	*GUX1*	AT	biallelic and monoallelic INDELs can be generated in the coniferous tree *P. radiata* using DNA and RNPs	[282]
*Populus davidiana × Populus bolleana*	CRISPR/Cas9	*PdbPDS1*	AT	Second, regeneration could produce homozygous mutant shoots at a high frequency and that kanamycin selection could increase the frequency of homozygous mutant shoots.	[266]
*Eucalyptus grandis x urophylla*	CRISPR/Cas9	*LFY*, *FT*	AT	The absence of male and female gametes and indeterminacy in floral development due to floral alteration because of disruption of ELFY function	[264]
*Populus alba × Populus glandulosa*	CRISPR/Cas12	*PDS*	AT	AsCas12a system is the most efficient and optimization of the co-cultivation temperature after Agrobacterium-mediated transformation from 22 to 28 °C to increase the Cas12a nuclease editing efficiency in poplar	[272]

AT = Agrobacterium-mediated transformation.

## 9. Conclusions

Due to the long rotation time of a forest plantation and the resulting long generation times necessary to complete a breeding cycle, the use of advanced methods with traditional breeding, such as high-throughput genotyping techniques, have been necessary, allowing the use of more precise approaches for determining the genetic architecture of traits of interest, such as genome-wide association studies and genomic selection. Moreover, the introduction of genome editing opens the door to new possibilities and perspectives in theoretical genetics and breeding science of forest trees and the fast remodeling of varieties. In this sense, mutations have greatly enhanced genetic resources for forest trees throughout the globe. With the development of TILLING as a high-throughput mutant screening method, stable gene-specific mutations are now extremely efficient. TILLING screening allows for more accurate detection of mutations at particular loci or genes. On the other hand, the development of new techniques such as CRISPR-based method has the potential to sustain productivity with less effort and cost substantially. More sophisticated techniques must be used to further complicate matters in selecting genome editing reagents and procedures for the regeneration of mutant plants. According to this, we believe that such problems will be adequately handled in the future.

## Figures and Tables

**Table 1:** Single nucleotide polymorphism (SNP) arrays developed for forest tree species. Genome size corresponds to the estimated genome size, N-SNP is the number of SNPs contained in each array. The density of markers is expressed as the number of SNPs per 1 Mb.

Species	Genome Size ^a^	N-SNP	Density (SNP/Mb) ^b^	Reference
*Eucalyptus* spp.	640 Mb	60 K	93.75	[81]
*Populus* spp.	420 Mb	34 K	91.5	[87]
*Populus nigra*	400–500 Mb	12 K	24–30	[95]
*Quercus* spp.	950–930 Mb	7.9 K	8	[89]
*Picea* spp.	20 Gb	7.3 K and 9.6 K	0.4–0.5	[88]
*Picea* spp.	20 Gb	50 K	2.5	[93]
*Araucaria angustifolia* (Bertol.) Kuntze	-	3 K	-	[90]
*Pinus* spp.	20–30 Gb	50 K	1.6–2.5	[91]
*Pinus radiata* D. Don	20–30 Gb	80–49 K	2–4	[96]
*Pseudotsuga menziesii* (Mirb.) Franco	16 Gb	28 K	1.75	[92]

^a^ Estimated size according to Ramos et al. (2018) [97], Zoldos et al. (1998) [98], Tuskan et al. (2006) [99], Kovach et al. (2010) [100], Zimin et al. (2014), (2017) [101,102], Neale et al. (2017) [103], Myburg et al. (2005) [104]. ^b^ Density estimated according to the N-SNP and Genome size.

**Table 2:** Published studies on genomic prediction (GS) in forest tree species in the last 5 years. Population, N-Markers and Model criteria correspond to the type of population used to implement GS, number of markers and the prediction models, respectively.

Species	Traits	Population	N-Markers	Model	Reference
*Eucalyptus pellita* F. Muell	DBH, HT, VOL	OP	19 K	GBLUP, BA, BB, BC, BL, BRR	[151]
*E. pellita* F. Muell	DBH, HT, VOL	OP	2 K	GBLUP, ssGBLUP	[153]
*E. robusta* Sm.	VOL, LIG, HCEL	Provenance trial	2.9 K	RKHS, GBLUP, EN	[154]
*E. benthamii* Maiden & Cambage	DBH, HT, VOL	OP	13 K	GBLUP, BA, BB, BC, BL, BRR	[151]
*E. nitens* (H.Deane & Maiden) Maiden	WD, DBH, TS, GST	OP	4.3 K	GBLUP	[26]
*E. nitens* (H. Deane & Maiden) Maiden	DBH, WD, WS, GST, TAS	OP	9.7 K	GBLUP	[155]
*E. nitens* (H. Deane & Maiden) Maiden	DBH, HT, ST and 9 wood related traits	OP	12 K	GBLUP	[152]
*E. urophylla x E. grandis*	HT, VOL, WD, PY, CBH	Go and G1	10 K	GBLUP, RRBLUP, BL, RKHS	[156]
*E. grandis × E. urophylla*	VOL, KL, HCEL, Wi, δ^13^C	Clones	3.3 K	GBLUP	[7]
*E. grandis × E. urophylla*	DBH, VOL, HT, MAI, CELL, S:G, LIG, WD	Full-sibs	33.4 k	ssGBLUP, GBLUP	[149]
*E. grandis*	DBH, HT, ST	OP	2.8 K	GBLUP multitrait	[157]
*E. grandis* W. Hill	FL, FW, CELL, S:G, WD, DBH, HT	Full-sibs	15 K	GBLUP	[65]
*E. globulus* Labill	BQ, DBH, ST, VOL, HT	Full-sibs and OP	14 K	RRBLUP, RRBLUPB, BA, BB, BL, PCR, SPCR	[13]
*E. globulus* Labill	HT, DBH, ST, BQ, PP	Full-sibs and OP	14 K	BA, BB, BC, BL, BRR	[36]
*E. globulus* Labill	VOL, WD	Clones	12 K	GBLUP, BL, BB, BC	[158]
*E. globulus* Labill	PP, ST, HT, DBH, BQ	Full-sibs	14 K	BRR, BL, BA, BB, BC, RKHS, GBLUP, DL, BRNN	[1]
*E. dunni* Maiden	DBH, ST	OP	11 K	ssGBLUP	[159]
*E. cladocalyx* F. Muell	HT, DBH, ST, SLD, PP, FI, BHT	OP	3.8 K	GSq, BA, BB, BC, BRR	[150]
*Picea glauca* (Moench) Voss	hat, DBH, VOL, AV, WD	Polycross, Full-sibs	4 K	GBLUP	[160]
*P. glauca* (Moench) Voss	HT, DBH, VOL, AV, PIC, PUN, PINC	Full-sibs	4.1 K	GBLUP	[161]
*P. mariana* (Mill.) Britton, Sterns & Poggenb	WD, DBH, HT, MFA	Full-sibs	5 K	GBLUP	[162]
*P. abies* (L.) H. Karst	HT, WD	OP	6.3 K	HBLUP	[163]
*P. abies* (L.) H. Karst	AV, WD, MFA, DBH, HT, SLD, WA	Polycross	4 K	GBLUP, BRR, BC	[164]
*P. abies* (L.) H. Karst	WD, MFA, MOE, AV	OP	130 K	GBLUP, BB, RKHS, RRBLUP	[64]
*P. abies* (L.) H. Karst	PP, AV, MOE, HT	Full-sibs	116 K	BLASSO, BRR, GBLUP, RKHS, BRR	[165]
*Pinus contorta* Douglas ex Loudon	WD, MFA, HT	Full-sibs and OP	19 K	GBLUP, BC	[166]
*Pinus radiata* D. Don	BCF, ST, ICH, ERB	Full-sibs and clones	67 K	GBLUP	[167]
*Pinus radiata* D. Don	ST, DBH, WD, MOE	Full-sibs	58.6 K	GBLUP	[155]
*Pinus sylvestris* Thunb.	HT, DBH, MFA, MOE, WD	Full-sibs	8.7 K	GBLUP, BRR, BL	[168]
*Hevea brasiliensis* Muell. Arg	RB	Full-sibs	0.3 K	RKHS, RRBLUP, BL	[169]
*H. brasiliensis* Muell. Arg	CBH (Two watering contrasting conditions)	Full-sibs	30 K	GBLUP	[170]
*Populus nigra* L.	HT, CBH, BF, BS, RST	Clones	8 K	GBLUP, BL	[171]
*Pseudotsuga menziesii* (Mirb.) Franco	JHT	Full-sibs	70 K	RRBLUP, GRR, BB	[32]
*Pseudotsuga menziesii* (Mirb.) Franco	HT, WD, DBH	Full-sibs	70 K	RRBLUP, GRR	[172]

AV: acoustic velocity; BB: Bayes B; BC: Bayes C; BF: bud flush; BHT: first, bifurcation height; BL: Bayesian LASSO; BQF: branch-cluster frequency; BRNN: Bayesian regularized neural network; BS: bud set; CELL: cellulose content; CBH: circumference at breast height; δ13C: stable carbon isotope composition; DBH: diameter at breast height; DL: Deep Learning; EN: elastic net methods; ERB: external resin bleeding; FI: flowering intensity; FL: fiber length; FW: fiber width; GRR: generalized ridge regression; GSq: combined method of GS and GWAS; GST: growth strain; HBLUP: genomic and pedigree-derived relationship matrix; HCEL: holo-cellulose; HT: tree height; ICH: internal checking; JHT: juvenile height; KL: Klason lignin; LIG: lignin content; MAI: mean annual increment; MFA: microfibril angle; MOE: modulus of elasticity; OP: Open-pollinated; PCR: principal components regression; PIC: picein concentration in needles; PICN: piceol concentration in needles; PP: pilodyn penetration; PUN: pungenol concentration in needles; PY: pulp yield; RB: rubber production; RKHS: Reproducing Kernel Hilbert Spaces; RRBLUPB: RRBLUP with variable selection procedure; RST: resistance to rust; S:G: syringyl and guaiacyl ratio; SLD: slenderness; SPCR: Supervised PCR; ssGBLUP: single-step GBLUP; ST: stem straightness; TAS: tangential air-dry shrinkage; VOL: volume; WA: weevil attack; WD: wood density; Wi: intrinsic water use efficiency; WS: wood stiffness.

## Data Availability

Data are contained within the article.
